# Unearthing novel fusions as therapeutic targets in solid tumors using targeted RNA sequencing

**DOI:** 10.3389/fonc.2022.892918

**Published:** 2022-08-10

**Authors:** Sungbin An, Hyun Hee Koh, Eun Sol Chang, Juyoung Choi, Ji-Young Song, Mi-Sook Lee, Yoon-La Choi

**Affiliations:** ^1^ Department of Health Science and Technology, Samsung Advanced Institute for Health Sciences & Technology (SAIHST), Sungkyunkwan University, Seoul, South Korea; ^2^ Laboratory of Molecular Pathology and Theranotics, Samsung Medical Center, School of Medicine, Sungkyunkwan University, Seoul, South Korea; ^3^ Department of Pathology, Severance Hospital, Yonsei University College of Medicine, Seoul, South Korea; ^4^ Department of Digital Health, Samsung Advanced Institute for Health Sciences & Technology Samsung Advanced Institute for Health Sciences & Technology (SAIHST), Sungkyunkwan University, Seoul, South Korea; ^5^ Department of Pathology and Translational Genomics, Samsung Medical Center, School of Medicine, Sungkyunkwan University, Seoul, South Korea

**Keywords:** fusion gene, solid tumors, next generation sequencing, diagnostics, targeted therapy, precision medicine

## Abstract

Detection of oncogenic fusion genes in cancers, particularly in the diagnosis of uncertain tumors, is crucial for determining effective therapeutic strategies. Although novel fusion genes have been discovered through sequencing, verifying their oncogenic potential remain difficult. Therefore, we evaluated the utility of targeted RNA sequencing in 165 tumor samples by identifying known and unknown fusions. Additionally, by applying additional criteria, we discovered eight novel fusion genes that are expected to process oncogenicity. Among the novel fusion genes, RAF1 fusion genes were detected in two cases. PTPRG-RAF1 fusion led to an increase in cell growth; while dabrafenib, a BRAF inhibitor, reduced the growth of cells expressing RAF1. This study demonstrated the utility of RNA panel sequencing as a theragnostic tool and established criteria for identifying oncogenic fusion genes during post-sequencing analysis.

## Introduction

Multiple fusions have been reported in tumors, several of which function as oncogenic drivers (1–3). With the development of next-generation sequencing (NGS), studies have mostly focused on identifying numerous novel fusions or elucidating the function of a single fusion gene in various tumors including cancer with diagnostic uncertainty (4–8). Further, as tumor-agnostic therapies have emerged as an option for cancer treatment, identification of genetic variations as well as histopathologic interpretation of cancer has gained increasing importance. Tumor-agnostic therapies target specific genomic alterations or molecular features regardless of tumor origin (9). For instance, although the morphologic spectrum of neurotrophic tyrosine receptor kinase (NTRK) fusion tumors was heterogenous, and included an undesignated tumor lineage, NTRK1-3 fusions in solid tumors were revealed as tumor-agnostic marker of response to treatment with a selective inhibitor (10). Therefore, detection of gene fusions based on accurate diagnosis can guide clinicians to establish appropriate therapeutic strategies.

Histopathological interpretation through microscopic features is still main evidence for tumor diagnosis. However, in some cases, diagnosis by histological findings is not possible because of poor differentiation or unknown tumor origin. To address this problem, guidelines for accurate diagnosis have been provided based on molecular marker detection; however, some cancers do not yet meet the criteria and cannot be diagnosed accurately. In cancers without defined molecular markers, NGS can be utilized to accurately diagnose as well as discover fusions in ambiguous tumors, thus providing new opportunities for patients who are not receiving appropriate treatment owing to diagnostic uncertainty.

Compared to other assays, targeted RNA sequencing is more effective in detecting fusions (11, 12). There are some sequencing methods used in clinical practice: whole exome sequencing-based capture probe method, the hybrid capture method, an amplicon-based method, and the anchored multiplex polymerase chain reaction (PCR) method. The whole exome sequencing-based capture probe method is good for detection known and unknown fusions but has limitation as a diagnostic method because of relatively low sequencing coverage depth and price (13). The hybrid capture method involves attaching a capture probe to the target gene, followed by sequencing. This method provides the advantage of capturing both known and unknown fusions but has the disadvantage of requiring a high input for sequencing. The amplicon-based method can perform sequencing with low input; however, it can only detect known fusions. Anchored multiplex PCR, which complements previous hybrid capture and amplicon-based methods, is a target enrichment method that uses gene-specific and universal primers. This method is advantageous for detecting known and unknown fusions with a low input (14).

Targeted RNA sequencing enables accurate detection of fusions through several fusion analysis pipelines. However, fusion detection accuracy may vary depending on the analysis criteria and the fusion callers used including Arriba, JAFFA, and STAR-Fusion (15). Therefore, most studies use a combination of multiple fusion callers to increase the reliability and accuracy of the analysis or develop additional analytical software for improved accuracy (16–18). Variations in outputs from different software yield varying results depending on the criteria set by the researchers; therefore, establishing standard criteria for fusion analysis is necessary.

In this study, we identified novel therapeutic fusions in tumors with diagnostic uncertainty using an exclusive RNA fusion panel and evaluated their oncogenic activity and therapeutic potency using *in vitro* assays. Subsequently, this study also suggests additional points for consideration in the identification of oncogenic fusions after RNA panel sequencing and demonstrates the utility of targeted RNA sequencing for fusion detection as a diagnostic tool.

## Materials and methods

### Sample collection

From 2010 to 2019, 195 formalin-fixed paraffin-embedded (FFPE) blocks from 195 patients and 23 frozen samples from patients with undifferentiated pleomorphic sarcoma, adenocarcinoma, rhabdomyosarcoma, and other solid cancers, which no driver mutations were previously found, were collected from Samsung Medical Center (SMC) in Seoul, Korea. Each sample was obtained from a different patient. Collection of patient samples was approved by the SMC Institutional Review Board (approval number 2019-05-141) and this study was performed in accordance with the Declaration of Helsinki.

### Panel design

The Cancer Gene-fusion by Multiplex PCR (CGMP) panel, a custom panel utilizing Archer AMP technology, comprised of 90 genes. It included the most frequent 16 genes in the quiver database (http://quiver.archerdx.com/), 53 genes included in the Archer FusionPlex Solid Tumor Kit (ArcherDX, Boulder, CO, USA), and 26 genes (five overlapping genes) in the Archer FusionPlex Sarcoma Kit. After the pilot test, 17 genes were included in the panel. Finally, sequencing was performed for 107 genes (**Supplementary Table 2**).

### RNA extraction and targeted sequencing

RNA was extracted from FFPE and frozen samples using an RNeasy FFPE Kit (73504; Qiagen, Hilden, Germany). Sample quality was assessed using a Nanodrop and Qubit 3.0 Fluorometer (Q33216; Thermo Scientific, Waltham, MA, USA). Libraries were prepared using an Archer library kit (SK0093; ArcherDX). Following the manual, we proceeded with PreSeq QC using a 10X VCP primer mix (ArcherDX, SA1026) after synthesizing first-strand cDNA. Second-strand cDNA was synthesized by selecting samples with VCP ct<31. Unidirectional gene-specific primers were used to enrich the target regions, followed by NGS on the Illumina MiSeq platform (San Diego, CA, USA). The resulting libraries were analyzed for the presence of relevant fusions. Reads were matched with a database of known fusions and other oncogenic isoforms. Samples with fewer than 10 unique RNA reads were classified as indeterminate and were excluded from further analysis. All analyzed fusions were in-frame and were predicted to have intact protein kinase domains or transcriptional factors. Mispriming and transcription readthrough events were excluded from the study. Fusions with a distance of less than 1 mbp between breakpoints, which is default value of fusion caller we used, were also excluded. After filtering, all remaining fusions were called and those that had not been reported previously were searched for across gene fusion databases (http://quiver.archerdx.com/, http://www.kobic.re.kr/chimerdb/,https://www.oncokb.org/ ) and named as novel fusions. All samples were confirmed for the presence of fusions using Arriba fusion caller (16). Samples were excluded if they were not called by either Archer or Arriba. All fusions were validated by IGV visualization and representative schematics of fusions were drawn using the Arriba script.

### RT-PCR

For fusion validation, cDNA was synthesized using SuperScript III Reverse Transcriptase (18080093; Invitrogen, Carlsbad, CA, USA) with 500 to 1000 ng of total RNA as the template. PCR was performed using fusion-specific primers (**Supplementary Table 5**). PCR product sizes were confirmed using 1% agarose gel electrophoresis. After validation, the products were purified using the QIAquick PCR Purification Kit (Qiagen, 28106) and sequenced.

### Identification of novel fusions

All structural variants, including in-frame gene fusions, translocations, and oncogenic isoforms resulting from deletions of whole exons, were subjected to further evaluation using the subsequent process. To exclude false-positive fusion candidates, a manual review was conducted. The fusions were classified as passenger fusions when the following conditions were met: (1) a fusion protein coding sequence that was out-of-frame; (2) the kinase domain was truncated, or the transactivation domain was eliminated from the predicted protein sequence; or (3) less than 10 split reads and 0 discordant mates were simultaneously found in the Arriba program; the 10 supporting reads used in the criteria means 10 unique fragments after removing duplicates. For each unique fragment, it resulted from the deduplication of 1 or more raw reads having the same molecular barcode. To confirm the novel fusions in tumors, gene-specific primers spanning the fusion junction in the transcript were synthesized, and the sequences of amplified products were confirmed using Sanger sequencing.

### PTPRG-RAF1 expression

An expression construct was prepared using all sequences from PTPRG exons 1 to 13 and RAF1 exons 7 to 18. Cloning was performed by subcloning two fragments into the pLenti6.3-V5-DEST vector (Invitrogen, V53306) using the sequence-and ligation-independent cloning method. The 293FT cell line from ATCC was transfected with the pLenti6.3/V5-PTPRG-RAF1 construct. The vector was placed into 293FT cells using the lentiviral kit (Thermo Scientific, K4975-00), and the virus was harvested and stored at -80°C. NIH3T3 cells were infected with lentivirus to create a stable line expressing the PTPRG-RAF1 fusion protein. Cells were cultured in DMEM supplemented with 10% fetal bovine serum and 1% antibiotic-antimycotic solution (Thermo Fisher Scientific).

### MTS assay

NIH3T3 empty vector cells (3T3 control cells) for negative control, and NIH3T3 PTPRG-RAF1-expressing cells (3T3 PR cells) (1x103 cells/well) were plated in a 96-well plate and allowed to settle overnight. After 24 h, the medium in each well was replaced with 10 µL of CellTiter 96 AQ One Solution reagent (G3582; Promega, Madison, WI, USA) in 90 µL of DMEM. The plates were incubated at 37°C for 4 h, in a humidified, 5% CO2 atmosphere. Absorbance was recorded at 490 nm using a Spectramax 190 plate reader (Molecular Devices, San Jose, CA, USA). The assay was carried out every 24 h for 96 h in triplicate. A t-test was performed to evaluate significant differences between the two groups.

### Spheroid culture

Aliquots of 100, 500, and 1000 3T3 control cells and 3T3 PR cells were plated in a 96-well round-bottom plate, and the growth of these cell lines was assessed. Five spheroids were measured in one experiment, and a total of three experiments were repeated. We measured both the long and short axes. Cell volumes were obtained by multiplying the square of the short- and the long-axis by two, and the volume change ratio was determined. A t-test was performed to evaluate significant differences between the two groups.

### Soft agar assay

The soft agar assay was performed as previously described (19). The base layer of each well consisted of 1.5 mL of serum-free 2X DMEM (Welgene, LM 201-50) with a final concentration of 0.5% Noble agar (BD Biosciences). After bottom agar solidification, 1.5 mL of 0.35% agar containing NIH3T3 cells (20,000) was seeded on the bottom agar layer and incubated for 14 days. Medium was changed every 2 days for 2 weeks. Colonies were fixed with 4% paraformaldehyde and then stained with 0.05% crystal violet (Sigma-Aldrich, C6158).

### Invasion assay

The matrigel coated 24-well Boyden chamber (Corning, 354480) with 8.0um PET membrane was used. For rehydration, we added culture media into bottom well and serum-free culture media into the chamber. The plate was incubated in 37°C for 2 hours. After removing the medium, we added culture media 500ul with 10% FBS into the bottom well and serum free media 500ul containing 5*10^4^ cells/well into the chamber. Cells were incubated for 24 hours in 37°C, 5% CO2 incubator. After the incubation, we scrubbed the surface of the camber using cotton tipped swabs. Cells were fixed with 70% ethanol for 10 min and stained with 0.2% crystal violet in 20% ethanol. Cells were counted in 4 fields of each membrane.

### Drug screening

The chip layout was designed to screen 12 compounds in a single micropillar array chip, as previously described (20). In this array, 80–100 cells were immobilized using 0.75% alginate. We tested 68 compounds in both 3T3 control and 3T3 PR cells. The 3T3 control cells and 3T3 PR cells (3 × 10^5^/well) were plated in 6-well plates and cultured in DMEM with DMSO and dabrafenib at concentrations ranging from 1 - 10 µM.

### Western blotting

Cells were harvested and lysed in RIPA buffer containing a protease inhibitor cocktail (P3100; GenDEPOT, Katy, TX, USA). Blots were probed using anti-c-Raf (1:1000, Cell Signaling Technologies, 9422S), anti-phosphoAkt (Ser473) (1:1000, Cell signaling, 9271S), anti-Akt(1:1000, Cell signaling, 9272S), anti-phospho-p44/42 MAPK (Thr202/Tyr204) (1:1000, Cell signaling, 9101S), anti-p44/42 MAPK (1:1000, Cell signaling, 9102S), anti–phosphoMEK 1/2 (1:1000, Cell Signaling Technologies, 2338S), and anti-β-actin (1:3000, sc-47778; Santa Cruz Biotechnology, Dallas, TX, USA).

## Results

### Development of CGMP panel

To detect fusions, we designed an anchored multiplex PCR-based NGS panel (Archer FusionPlex) with 90 genes including kinase and transcription factor genes in solid tumor such as lung adenocarcinoma, lung squamous cell carcinoma, and soft tissue sarcoma. The primary fusion panel was validated using 21 FFPE samples (**Supplementary Figure 1** and **Supplementary Table 1**). One sample failed sequencing quality control because of a low gene-specific primer (GSP) percentage. Fifteen out of the 20 samples showed the presence of fusions and 14 different fusions were detected. The fusion information of RT-PCR or fluorescence *in situ* hybridization (FISH) results were available for 11 samples of the 15 fusion-positive samples. The panel sequencing results of 11 samples matched with RT-PCR or FISH results and four different fusions were additionally detected in four samples using our panel. The primary panel did not detect any fusions in the remaining five samples; one sample had a previously reported DDIT3 rearrangement detected in FISH but was not detected in our study because DDIT3 was not included in our panel. To complement the initial sequencing results, the panel was updated to include cancer-related genes containing the DDIT3 gene. (**Supplementary Table 2**). Finally, we generated a targeted sequencing panel named Cancer Gene-fusion by Multiplex PCR (CGMP), which captured 107 genes in total.

### Identification of fusions in diagnostically uncertain tumors

**Figure 1:**
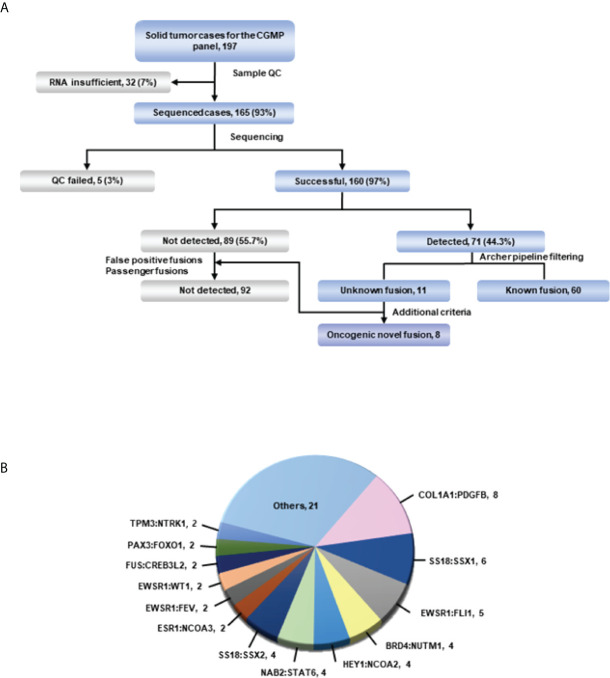
Overview of targeted sequencing. **(A)** Schematic of the process. All fusions were detected in the Archer pipeline. The green line represents confirmation of detected fusions with the Arriba fusion caller. **(B)** Fusions and their count detected in more than 2 cases in targeted RNA sequencing. Fusions from only one case were included in others.

To determine the presence of fusions in solid tumors with diagnostic uncertainty, we investigated 165 samples using CGMP panel sequencing. We removed false positive results which were mispriming or readthrough events through manual review. In total, 160 samples were successfully sequenced, and fusions were detected in 71 samples (**Supplementary Table 3** and **Figure 1A**). Three samples were excluded because of the additional criteria applied in the filtering process, leaving a total of 68 fusion-positive samples with 34 fusions; of these, thirteen fusions were detected in two or more samples. COL1A1-PDGFB was most frequently identified in eight samples (**Figure 1B**; **Supplementary Table 3**). After analyzing the sequencing data using the Archer pipeline, 12 unknown fusions were detected in 13 samples. However, the ESR1-NCOA3 fusion was found to be previously reported as an oncogenic fusion in adenosarcoma (21, 22) and was excluded from the novel fusions. Overall, we identified 11 novel fusions from 160 samples. Subsequently, we manually reviewed these novel fusions to remove passenger fusions which are real fusion but not clinically significant. The oncoprotein function of the novel fusion was confirmed through the following criteria: 1) whether functional domains such as the protein kinase domain or transcription activation domain are retained; 2) whether the fusion is an in-frame gene fusion; and 3) whether the read count of the fusion transcript is 10 or more. If these criteria were not met, the genes were excluded as passenger fusions. The SS18-GREB1 fusion was identified in sample AF0050 from a 59-year-old woman diagnosed with undifferentiated uterine sarcoma. However, SS18-GREB1 fusion was excluded from oncogenic novel fusions because the transcription activating QPGY domain, a functional domain of SS18 gene was eliminated by gene rearrangement (23, 24). Two fusions (PDZD2-AKT2 and KIF13A-PIK3CA) were also excluded after checking the reading frame and read counts. A PDZD2-AKT2 fusion detected in sample AF0162, which was diagnosed as carcinosarcoma, was excluded because the intronic fusion led to out-of-frame to eliminate the function of AKT2 and low read counts (<10). A KIF13A-PIK3CA fusion in sample AF0201 with non-small cell carcinoma was excluded because an out-of-frame mutation was found to eliminate the function of PIK3CA. Finally, novel fusions (PLAGL1-FOXO1, PTPRG-RAF1, FOS-GLI1, MAZ-NCOA2, SS18-KLF14, ZNF462-MUSK, LSM1-NRG1, and APPL2-RAF1) were identified in eight samples (**Figure 1A**, **Table 2**; **Supplementary Table 3**). The expression of fusions was confirmed by RT-PCR using fusion-specific primers (**Supplementary Table 5**).

### Evaluation of diagnostic utility of fusion detection in cancers

To confirm the correlation between fusions and clinicopathological characteristics, we classified our samples into 14 groups. We largely classified the cases into two categories, clear diagnosis and descriptive diagnosis. When the case meets the diagnostic criteria of ‘WHO Classification of Tumors’, we gave a clear diagnosis using the term that ’WHO Classification of Tumors’ defines. However, when the histological findings of the case are equivocal or unmet to diagnostic criteria of any disease which ’WHO Classification of Tumors’ suggests, a descriptive diagnosis such as ‘Atypical myxoid spindle cell neoplasm’ was used (25) and randomly assigned numbers to groups (**Table 1**; **Supplementary Tables 3**, **4**). In group 7, we detected fusions in 37 of 42 patients (88%); this group exhibited the highest frequency of fusion among the classes of sarcoma. In group 10, where sarcoma was frequently observed with neurogenic differentiation, fusions were detected in three of five (60%) patients. In group 1, adenocarcinoma, fusions were detected in 5 of 9 (57%) patients (**Table 1**).

**Table 1:** Detection of fusions according to pathologic classification.

Group	Number of patients	Number of detected fusions	Frequency	Detected Fusions	Number ofSamples
1	9	5	56%	TPM3-NTRK1	2
				EML4-ALK	1
				KIF5B-RET	1
				SLC34A2-ROS1	1
2	3	0	0%		
3	4	3	75%	BRD4-NUTM1	2
				WHSC1L1:NUTM1	1
4	3	0	0%		
5	13	2	15%	ESR1-NCOA3	2
6	56	13	23%	COL1A1-PDGFB	1
				EWSR1-FLI1	2
				JAZF1-NONE-PHF1	1
				BRD4-NUTM1	1
				ZNF462-MUSK*	1
				EWSR1-WT1	1
				COL1A1-PDGFB	1
				LSM1-NRG1*	1
				SS18-SSX2	1
				APPL2-RAF1*	1
				FUS-NFATC2	1
				MAZ-NCOA2*	1
7	42	37	88%	SS18-SSX1	6
				COL1A1-PDGFB	4
				NAB2-STAT6	4
				EWSR1-FLI1	3
				HEY1-NCOA2	3
				EWSR1-FEV	2
				FUS-CREB3L2	2
				PAX3-FOXO1	2
				SS18-SSX2	2
				CAPZA2-MET	1
				CIC-DUX4	1
				EWSR1-CREM	1
				EWSR1-NR4A3	1
				EWSR1-WT1	1
				FUS-ERG	1
				SS18-KLF14*	1
				TAF15-NR4A3	1
				TFE3-ASPSCR1	1
8	9	0	0%		
9	11	3	27%	COL1A1-PDGFB	2
				BRD4-NUTM1	1
10	5	3	60%	PTPRG-RAF1*	1
				SS18-SSX2	1
				FOS-GLI1*	1
11	4	1	25%	HEY1-NCOA2	1
12	1	0	0%		
13	1	0	0%		
14	4	1	25%	PLAGL1-FOXO1*	1

*Novel fusion partners identified using CGMP panel sequencing

Group number; 1. Adenocarcinoma 2. Carcinoma, a known lineage 3. Carcinoma, a known fusion 4. Carcinoma, unknown lineage; undifferentiated carcinoma 5. Uterine malignancy 6. Sarcoma, unknown lineage; undifferentiated sarcoma 7. Sarcoma, known fusion 8. Sarcoma, myogenic lineage without known fusion 9. Sarcoma, fibroblastic lineage without known fusion 10. Sarcoma, neurogenic lineage without known fusion 11. Sarcoma, chondroid or bone lineage without known fusion 12. Sarcoma, lipogenic lineage without known fusion 13. Sarcoma, vascular or perivascular lineage without known fusion 14. Other malignancy.

**Figure 2:**
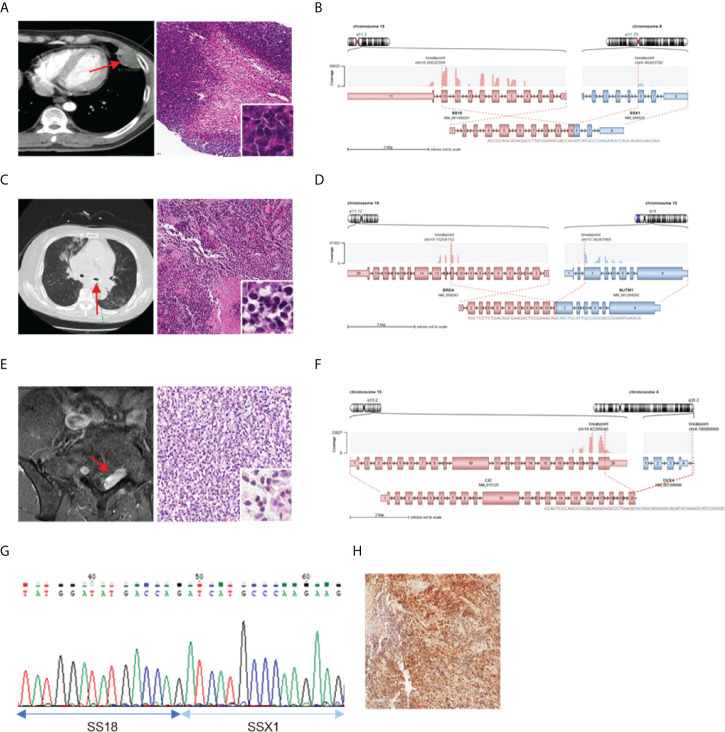
Cases with complemented diagnosis through targeted sequencing. **(A)** Chest computed tomography image of 47mm sized pleura based soft mass lesion (left) and H&E staining of tissue diagnosed with Ewing sarcoma (right). **(B)** Schematic of the SS18-SSX1 fusion. **(C)** CT image of mediastinum (left) H&E staining of tissue diagnosed as undifferentiated carcinoma (right). **(D)** Schematic of the BRD4-NUTM1 fusion. **(E)** Magnetic resonance imaging of spine (left) and H&E staining of tissue diagnosed undifferentiated small round cell (right). **(F)** Schematic of the CIC-DUX4 fusion **(G)** Confirmation of SS18-SSX1 fusion with RT-PCR and Sanger sequencing. **(H)** NUT IHC. *Red arrows indicated tumor legion.

Further investigation was conducted to determine the correlation between the identified fusions and clinicopathological characteristics of fusion-positive samples. In most cases where a known fusion was detected, the clinicopathologic features of the tumors and the previously known reports were found to be identical; however, there were three cases in which they did not match. In one case (AF0052), the characteristics of the fusion and the clinicopathological features were completely different; in the remaining two cases (AF0065 and AF0154), a different diagnosis was made because the genetic test for fusion detection was not performed. Sample AF0052 was obtained from a 42-year-old man, who complained of left pleuritic pain. Chest computed tomography (CT) revealed a 4.7-cm pleura-based soft lesion in the anterior aspect of the left hemithorax. Histologically, a cellular tumor comprising malignant oval to spindle cells with necrosis was observed (**Figure 2A**), which was positive for Bcl-2, CD99, and FLI-1, but negative for pan-cytokeratin, STAT6, and CD34. FISH analysis for EWSR1 was negative but the patient was diagnosed with Ewing sarcoma based on histological findings. We identified the SS18-SSX1 fusion (**Figure 2B**), as a diagnostic marker in this case, which was confirmed by RT-PCR and Sanger sequencing (**Figure 2G**). Therefore, the patient was diagnosed as having synovial sarcoma.

Sample AF0065 was obtained from a 75-year-old woman diagnosed with small-cell lung carcinoma with adrenal and bone metastases. Chest CT revealed extensive lymphadenopathy in the mediastinum without a definite primary focus (**Figure 2C**). A biopsy specimen was diagnosed as a poorly differentiated carcinoma with suspected small-cell carcinoma. The tumor cells were focally positive for synaptophysin and pan-cytokeratin, but negative for CD56 and chromogranin. A tracheal biopsy using bronchoscopy revealed undifferentiated small malignant cells with some large cells (**Figure 2C**). Tumor cells were weakly positive for synaptophysin and showed a 95% Ki-67 index; therefore, small-cell carcinoma with some large cells was more likely. However, from the sequencing data, a BRD4-NUTM1 fusion was identified in the tumor (**Figure 2D**), and the diagnosis was changed to NUT carcinoma. IHC, RT-PCR, and Sanger sequencing were then performed for further verification (**Figure 2H**).

AF0154 was obtained from a 57-year-old man, who complained of left thigh pain and left toe numbness. Magnetic resonance imaging (MRI) of the spine revealed an enhancing mass in the S1 epidural space, suggestive of a neurogenic tumor (**Figure 2E**). The results of IHC were as follows: positive for FLI-1, CD99, and vimentin; negative for synaptophysin and desmin; normal expression of INI-1; and a Ki-67 labeling index of 73%. We implemented EWSR1 FISH because we suspected Ewing sarcoma; however, the tumor was negative for EWSR1 translocation. Therefore, because there was absence of marker for diagnosis, the patient was diagnosed with small round cell sarcoma. However, upon sequencing, a CIC-DUX4 fusion was identified in the tumor (**Figure 2F**).

### Clinicopathologic features of novel fusions in clinical samples

**Figure 3:**
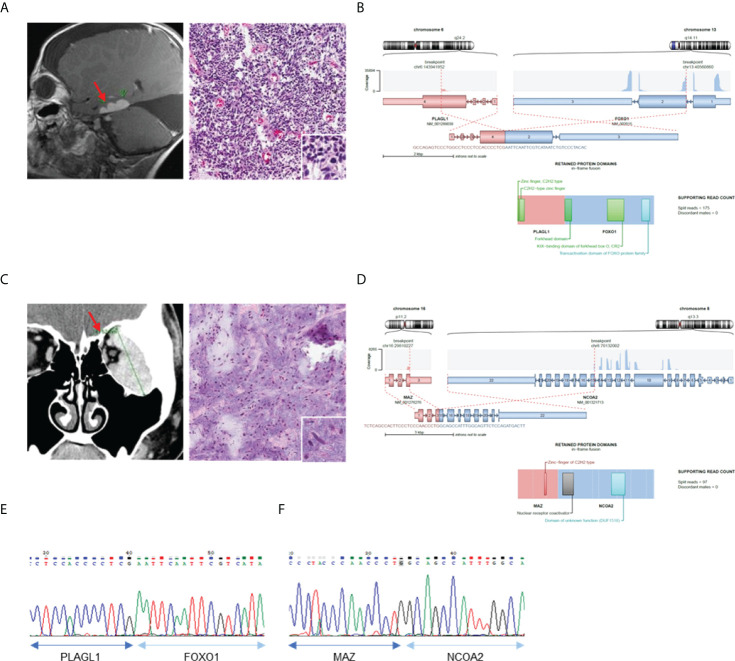
Confirmation of novel fusions. **(A)** MRI brain image show tumor in left temporal lobe (left) and H&E staining of tumor tissue (right). **(B)** Schematics of *PLAGL1-FOXO1* and remaining protein domains of fusion. **(C)** MRI of left orbit with myxoid soft tissue lesion (left) and H&E staining of myoepithelioma (right). **(D)** Schematics of *MAZ-NCOA2* and remaining protein domains of fusion. **(E)** Confirmation of *PLAGL1-FOXO1* fusion expression using RT-PCR. **(F)** Confirmation of *MAZ-NCOA2* fusion expression using RT-PCR *Red sparrow indicate tumor legion.

We then investigated the clinicopathological features of eight cases in which novel fusions were detected (**Table 2**). Except for one 74-year-old woman, all other patients were under the age of 50 years. The histologic features mainly consisted of spindle cell tumors, but most of them were diagnostically uncertain. Two cases with novel fusions were examined in more detail. The first case (AF0033) was a 3-year-old male patient diagnosed with an embryonal tumor, not otherwise specified. MRI revealed metastatic or recurrent leptomeningeal lesions in the brain, comprising undifferentiated or primitive small round and spindle cells, with diffuse neuronal and focal glial differentiation (**Figure 3A**). The PLAGL1-FOXO1 fusion was discovered through sequencing. The DNA-binding domain (DBD) of PLAGL1 and the transactivation domain of FOXO1 remained intact (**Figure 3B**) in the fusion protein. The fusion transcript was confirmed using RT-PCR (**Figure 3E**). The second case (AF0112) was a 17-year-old woman, who complained of a protruding left eye. The MRI reports from a different hospital showed a large irregular mass involving her left orbit; microscopically, it contained oval to spindle cells with mild atypism in confluent myxoid stroma, which formed a vague cord-like structure (**Figure 3C**). The MAZ-NCOA2 fusion was detected using our panel. The remaining protein domains, the DBD of MAZ, and the transactivation domains of NCOA2 were intact (**Figure 3D**). The fusion transcript expression was identified using RT-PCR (**Figure 3F**). We also evaluated the MRI results and cell morphology of other samples with novel fusions, and we predicted the activation of PK genes (ZNF462-MUSK) through enhancer hijacking or oncogenic gene expression by transactivation with TF genes (FOS-GLI1, SS18-KLF14, LSM1-NRG1), using our panel sequencing analysis (**Supplementary Figure 2, 3**).

**Table 2:** Clinical characteristics of the cases in which novel fusions were detected.

Case	Age	Sex	Fusion	Primary site	Metastasis site	Diagnosis	Histology	Split read count	Discordant read count	IHC
AF0033	3	M	PLAGL1-FOXO1	Brain	Leptomeninges	Embryonal tumor, NOS	Undifferentiated or primitive small round and spindle cells and diffuse neuronal and focal glial differentiation	175	0	Desmin(-)
AF0062	49	M	PTPRG-RAF1	Sacrum	Lung	MPNST	Spindle proliferation in myxoid stroma	87	5	STAT6(-) TLE-1(-) EMA(-) calponin(-) H3K27me3(-) S-100(-) MUC4(-)
AF0111	28	F	FOS-GLI1	Abdominal wall muscle		Myxoid neurogenic tumor	Spindle cells with moderate cellularity and pleomorphism	110	0	S-100(+) CD99(-) CD34(-) D2-40(-) synaptophysin (-) NSE(-) CD10(-)
AF0112	17	F	MAZ-NCOA2	Orbit		Myoepithelioma	Spindle cells with mild atypism in confluent myxoid stroma	97	0	S-100(+) Pan-CK(-) p53(-) CD34(-)
AF0140	40	M	SS18-KLF14	Psoas muscle	Pelvic wall	Synovial sarcoma	Monotonous spindle cell forming fascicular pattern	151	21	S-100(-)
AF0171	40	M	ZNF462-MUSK	Small bowel		Myxoid sarcoma	Atypical scattered spindle cells in the myxoid background	37	3	Desmin(+) MDM2(+) S-100(-) smooth muscle actin (-) c-KIT(-)
AF0197	74	F	LSM1-NRG1	Heart	Thoracic spine	UPS	Oval to spindle tumor cells and some scattered pleomorphic cells with focal necrosis	11	0	MDM2(+) CD31(-) CD34(-)
AF0216	23	F	APPL2-RAF1	Mandible	Lung	UPS	Moderately pleomorphic spindle cells with monotonous morphology and high cellularity	87	5	Desmin(-) S-100(-) CD34(-) smooth muscle actin(-) STAT6(-)

NOS, not otherwise specified; MPNST, malignant peripheral nerve sheath tumor; UPS, undifferentiated pleomorphic sarcoma.

**Figure 4:**
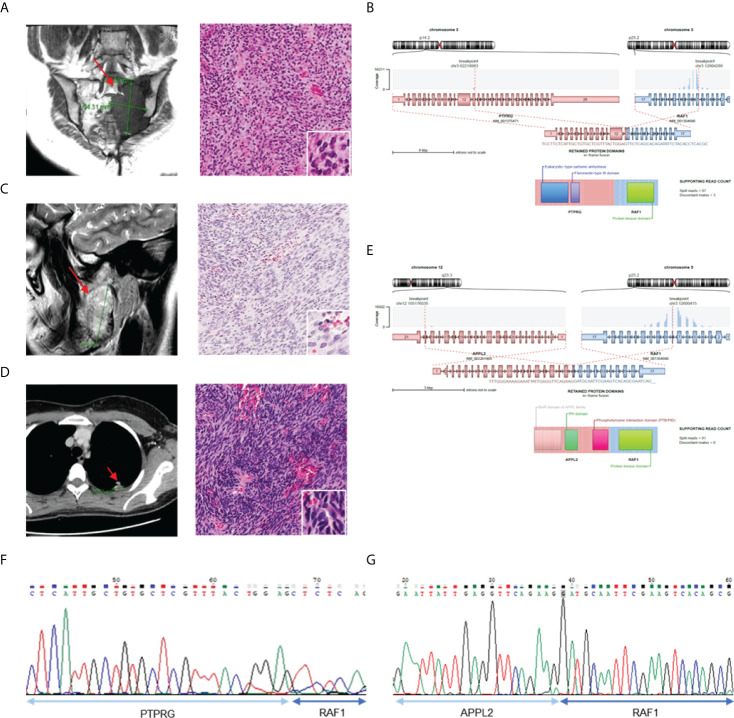
Validation of the cases with RAF1 gene fusion. **(A)** MRI image show tumor in left sacrum (left) and H&E staining of tumor tissue (right). **(B)** Schematics of *PTPRG-RAF1* and remaining protein domains of fusion. **(C)** MRI of right mandible with 5.3 sized mass (left) and H&E staining with pleomorphic spindle cells with monotonous morphology (right). **(D)** CT of chest shows metastatic tumor in left upper lobe (left) and H&E staining of tumor tissue (right). **(E)** Schematics of *APPL2-RAF1* and remaining protein domains of fusion. **(F)** Confirmation of *PRPRG-RAF1* expression using RT-PCR. **(G)** Confirmation of *APPL2-RAF1* expression using RT-PCR.

We found two cases (AF0062 and AF0215) in which the RAF1 gene had novel fusion partners (**Figure 4**). All RAF1 fusions had retained the RAF1 kinase domain and in-frame protein coding sequence, as well as showed sufficient read counts (87, 91). In AF0062, the first RAF1 fusion-positive case, a 49-year-old woman was referred to the hospital for a recurrent tumor in the left sacrum, which was diagnosed as a solitary fibrous tumor at a different hospital. MRI after surgery showed an interval increase in the size of the residual sacral tumor. We reviewed the histology of the pelvic mass, which showed spindle cell proliferation in the myxoid stroma (**Figure 4A**). Based on the histological morphology and loss of H3K27me3 expression, the patient was diagnosed with malignant peripheral nerve sheath tumor (MPNST). Using CGMP panel sequencing, we identified a PTPRG-RAF1 fusion (**Figure 4B**). This novel fusion expression was evaluated by RT-PCR (**Figure 4F**). In the second case, AF0215, a 23-year-old woman, experienced pain, and neurologic symptoms around her right chin. The MRI showed a 5.3 cm sized mass involving the ramus and condyle of the right mandible, and the tumor was microscopically composed of moderately pleomorphic spindle cells with a nearly monotonous morphology and high cellularity (**Figure 4C**). Three years after surgery for the primary tumor, surgical resection of the recurrent tumor was performed at the same site. One year later, metastatic tumors were found in the lower and upper lobes of the left lung (**Figure 4D**). Tests for SS18-SSX1 gene rearrangement and FISH, under the possibility of synovial sarcoma, were found to be negative. The sample was included in this study and CGMP panel analysis identified the APPL2-RAF1 fusion (**Figure 4F**). The fusion was confirmed by RT-PCR (**Figure 4G**). Two years later, the patient had again metastasized to the lungs, and the same APPL2-RAF1 fusion was detected by NGS. Given that the APPL2-RAF1 fusion was also detected using RT-PCR from the primary and recurrent tumor tissue in the mandible, it is reasonable to assume that it was the oncogenic driver since the initial tumor development.

### Functional characterization of PTPRG-RAF1

**Figure 5:**
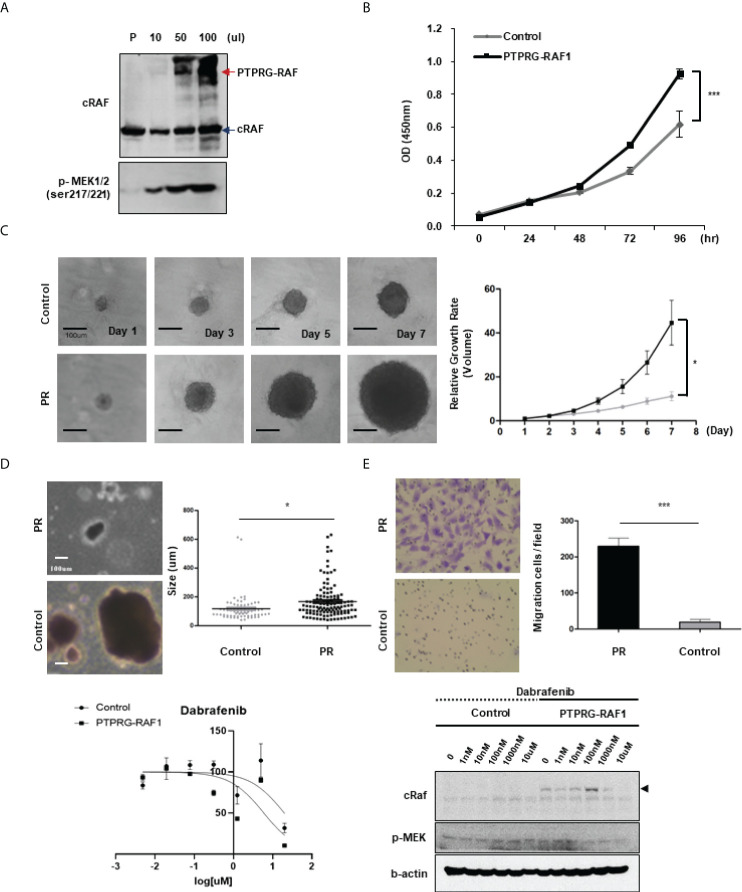
Oncogenicity of *PTPRG*-*RAF1*. **(A)** Western blotting showing RAF1 expression in the parental cell line (293FT), with an PTPRG-RAF1 fusion of 140 kDa and phospho-MEK1/2 expressed in 293FT cells transfected with the *PTPRG*-RAF1 expression plasmid. **(B)** Proliferation of NIH3T3 fusion-expressing cells (PR cells) by MTS assay (p<0.001). **(C)** Proliferation of PR cells assessed in spheroid culture, and relative growth rate in 3D culture condition. Clones were taken images and measured the diameters, and the volume was calculated using the formula described in the method (p<0.0001). **(D)** *In vitro* transforming assay in soft agar. NIH3T3 control and PR cells were cultured for 14 days in soft agar. The size of colonies was measured. *p < 0.05. **(E)** Invasion assay conducted in control and PR cells (p<0.001) **(F)** High throughput screening for antiproliferative activity; only dabrafenib showed a response (IC_50_ approximately 1 µM). **(G)** Effect of dabrafenib treatment in PR expressing cells. Cells were treated with the indicated concentrations of dabrafenib and after 72 h, cells were lysed and expression of the indicated proteins was assessed by western blot. The experiments were performed in triplicate with similar results. Error bars represent standard deviations of the means.

The RAF1 fusion activated by the RAF1 (CRAF) gene has been reported in a few melanomas and pediatric low-grade gliomas (26, 27); in some cases, MEK inhibitors have been reported to be effective against RAF1 fusions (5, 27, 28). Therefore, to determine whether the RAF1 fusions detected in our samples have oncogenic function and can be considered potential targets, we investigated the oncogenic effects of the PTPRG-RAF1 fusion. To introduce the PTPRG-RAF1 fusion into cells, we constructed a pLenti vector containing the PTPRG-RAF1 gene and used it to infect the 293FT cells. The RAF1 fusion protein was then expressed in 293FT cells in a dose-dependent manner without affecting endogenous RAF1 expression; in addition, the phosphorylation of MEK1/2, a downstream signal of RAF1, was augmented in the same manner (**Figure 5A**). We also observed that MAPK and PI3K pathway was activated by PTPRG-RAF1, by confirming the elevated levels of phospho-Akt and phospho-pErk in NIH3T3s (**Supplementary Figure 4**). To validate the oncogenicity of PTPRG-RAF1 fusion, we generated a stable cell line using NIH3T3 cells and investigated whether cell growth was increased by the fusion expression. We showed that the relative cellular growth rate was increased by approximately 49% compared with that in the parental cell line at 96 h (p <0.001; **Figure 5B**). To confirm cellular growth in a three-dimensional (3D) environment (29), 3T3 control and PR cells were cultured in round bottom plate and the clonal diameters were measured from images obtained every other day for 7 days. On the 7th day, after comparing the diameters of clones, we found that the clones of the PR cells had a statistically significant increase in the relative growth rate using volumetric measurements compared with the negative clones (p<0.05; **Figure 5C**). We also evaluated cellular transformation by performing soft agar assay, NIH3T3 PR cells formed significantly larger colonies compared with vector controls (**Figure 5D**, p=0.03). We also focused on the fact that all cancers with RAF1 fusion had lung metastasis and investigated whether fusion affects the upregulation of cell invasion by Matrigel invasion assay. As a result, cellular invasion was enhanced compared to the control (p=0.0007, **Figure 5E**). In a previous study, the susceptibility of RAF1 gene fusion to MEK inhibition has been reported. To investigate drugs that inhibits the growth of the PR cells, high-throughput screening using 66 drugs approved for targeted therapy was performed. Among drugs, the MEK inhibitor, trametinib and AZD6244, did not inhibit the growth of PR cells, whereas the BRAF kinase inhibitor, dabrafenib, showed inhibition of cell growth (IC50 value 1.25 µM) (**Figure 5F**). Using an *in vitro* growth assay, we observed that the inhibitory effect of dabrafenib in PR cells at doses ranging from 0.1-1 µM. (**Supplementary Figure 5**). Dabrafenib moderately inhibited the growth of PR cells compared with the parental cells at above 0.1 µM and induced a decrease in PTPRG-RAF1 expression and MEK1/2 phosphorylation (**Figure 5G**). Taken together, we confirmed that PTPRG-RAF1 fusion is an oncogenic driver gene that increases tumor proliferation and can be targeted for cancer therapy.

## Discussion

Histopathological features and protein expression patterns of tissue specimens are used to provide an accurate and definitive diagnosis. However, certain tumors such as undifferentiated pleomorphic sarcoma are histopathologically ambiguous, meaning that they cannot be specifically diagnosed even after considering all available pathological approaches. To avoid this ambiguity, new standards have been established and applied for pathological diagnosis (30). However, diagnostic confusion still arises among pathologists and clinicians, and such instances are referred to as ‘undifferentiated,’ ‘poorly differentiated,’ ‘uncertain,’ or ‘undefined.’ In such cases, patients may be excluded from receiving appropriate care because of organ-based diagnosis and treatment. Herein, we conducted targeted RNA fusion panel sequencing using 39 ambiguous tumor samples diagnosed as “undifferentiated” or “unclassified” in total 165 tumor samples and identified ten oncogenic fusions (**Supplementary Table 3**). The results of fusion detection in approximately 26% of the patients confirmed the importance of identifying genetic variations as well as histopathologic interpretations in cancer and showed that identification of genetic variations could facilitate appropriate treatment of patients.

Oncogenic fusions have been identified from various solid tumors without being limited to specific cancer types. In this study, we tried to identify oncogenic driver genes using a targeted fusion panel without limiting our screening to specific cancer types. We identified 26 known fusions in 60 samples and detected three cases that were misdiagnosed. We also performed an analysis using additional criteria to predict the oncogenic effects of novel fusions. Finally, we identified eight novel pathogenic fusions. Furthermore, we showed that the PTPRG-RAF1 fusion has oncogenic potential through an *in vitro* assay. We examined the potency of tumor-agnostic therapy for fusion-driven cancer, by assessing the effect of BRAF inhibitor in RAF1 fusion-driven cancer. Overall, this study provides guidelines on how to accurately diagnose and find appropriate treatment options for tumors with ambiguous diagnoses, which cannot be determined through single assays in the clinic.

We retrospectively reviewed the cancer samples; therefore, except for two cases, AF0052 and AF0152, our sequencing data were not directly used for clinical diagnosis (**Figure 2**). In the case of AF0052, the tumor recurred; the histological findings for the recurrent tumor were the same as those for the initial surgical specimen, but the samples were negative for EWSR1 and SYT in FISH analysis. Based on the NGS results, we performed RT-PCR and found a SS18-SSX1 fusion in the tumor. Eventually, we revised the diagnosis of synovial sarcoma. In the case of AF0152, approximately two years after tumor removal, a 3 cm tumor recurred at the surgical site. External consultation indicated that the tumor cells were focally positive for WT1 and ETV4, consistent with CIC-rearranged sarcoma; however, CIC FISH did not identify the CIC gene rearrangement. In this patient, the CIC-DUX4 fusion was confirmed using CGMP panel sequencing, which could have altered the diagnosis. Based on these results, we demonstrated that errors from the previous single gene assays could be supplemented by panel sequencing, and that the diagnosis reliability can be improved.

Although the use of NGS in clinical diagnosis is widely accepted, a few considerations are necessary for supplementing a diagnosis with NGS. First, it is necessary to determine whether a panel is suitable for diagnostic purposes. In case of AF0215, the fusion was not detected in the recurrent tumor because the initially used NGS panel was not fusion specific. However, the APPL2-RAF1 fusion was detected retrospectively in metastatic tumors using the CGMP panel and other fusion specific NGS panels. The purpose of each NGS platform is different; therefore, it is necessary to select an appropriate NGS platform. Next, the presence of a genetic variable outside target region should be considered when performing targeted sequencing. To identify fusions that were not detected in the CGMP panel, we selected 30 fusion-negative samples for RNAseq (data not shown), and found that no fusions were detected in 29 cases, but a novel FMNL3-LRRK2 fusion was identified in the intimal sarcoma sample, AF0048.

**Figure 6:**
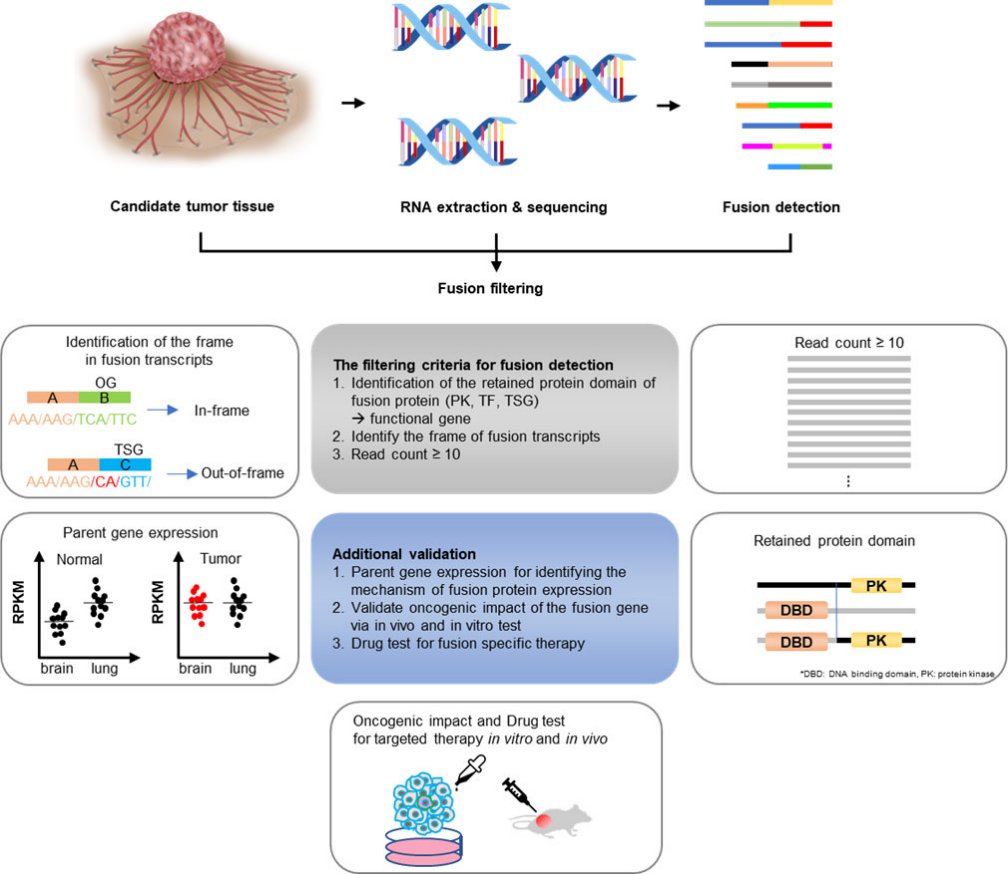
Gene fusion detection workflow. If the fusions are expected to be present in cancers whose exact cause is not determined using the existing diagnostic methods, RNA was extracted from the tissues and targeted sequencing was conducted. The sequencing data were first analyzed using the Archer pipeline. Candidate fusions were subsequently filtered further by gene distance, gene function, frame, and read count. Additional analyses were conducted, including checking for intact functional protein domains and the expression of parent genes in the tumor. Finally, a fusion expression plasmid was produced to conduct *in vitro* and *in vivo* experiments.

There are several criteria for predicting whether a fusion is oncogenic or not. First, when analyzing the reading frame, if the tumor suppressor gene forms an out-of-frame fusion, it could be oncogenic due to loss-of-function (31). The CGMP panel mainly uses kinase and transcription factor genes; therefore, only in-frame fusion can be detected. Next, by identifying the remaining functional protein domains, such as the protein kinase domains or transactivator domains, the read counts could result in the exclusion of cases where there is no remnant protein domain of the functional gene, or the read count is low. Additionally, the RNA expression of each of the genes that make up fusions may be upregulated because of enhancer hijacking or other processes, indicating the oncogenicity of the fusion. Therefore, RNA expression was confirmed through the Archer pipeline (**Figure 6**). The supervision in this study was performed manually; however, with the development of a systematic analysis method, the analysis of gene fusions and their utility in cancer diagnosis can be improved.

RAF1 fusions are actionable molecular events that have been reported in various tumor types, including melanoma, invasive ductal carcinoma, and lung adenocarcinoma. We discovered novel RAF1 fusions in two cases of malignant peripheral nerve sheath tumor and undifferentiated pleomorphic sarcoma. In cases of the APPL2-RAF1 fusion, RAF1 inhibitor use is recommended if metastatic relapse occurs, because the fusion was continuously detected from three recurrence and metastatic tumors after the initial tumor was identified. We showed that the RAF1 fusion detected using the CGMP panel has therapeutic potential *via* MEK/MAPK signaling activation and that dabrafenib, a BRAF inhibitor, was effective against PTPRG-RAF1 expressing cell lines *in vitro*. Therefore, fusion detection could be a practical diagnostic and therapeutic tool, considering that MEK inhibitors, such as dabrafenib, are effective against RAF1 fusion-harboring tumors (5). Through a series of experiments, we thus demonstrated that a novel fusion could be an oncogenic driver gene of tumors with diagnostic uncertainty as well as serve as a therapeutic target.

In conclusion, if a tumor is undiagnosed or its therapeutic target is not identified, a series of fusion searches using RNA NGS, elaborate analyses, and *in vitro* validation can offer possible oncogenic targets. This could be useful in making an accurate diagnosis and providing suitable treatment options for patients as well as for understanding the biology of fusion-driven cancer.

## Data availability statement

The datasets presented in this study can be found in online repositories. The names of the repository/repositories and accession number(s) can be found below: https://www.ncbi.nlm.nih.gov/sra/PRJNA816260, PRJNA816260.

## Ethics statement

The studies involving human participants were reviewed and approved by SMC Institutional Review Board (approval number 2019-05-141). Written informed consent to participate in this study was provided by the participants’ legal guardian/next of kin. Written informed consent was obtained from the individual(s), and minor(s)’ legal guardian/next of kin, for the publication of any potentially identifiable images or data included in this article.

## Author contributions

YLC conceived the original idea. YLC and MSL supervised the study. SA and JYS carried out sample preparation and designed the panel. HHK collected the clinical samples and co-wrote the manuscript. ESC and JC critically reviewed the study’s proposals. SA performed the analysis of sequencing data and co-wrote the manuscript. All authors provided critical feedback and helped shape the research, analysis, and manuscript.

## Funding

This work was supported by National Research Foundation of Korea (NRF) grants funded by the Korean government (Ministry of Science, ICT, and Future Planning) (nos. 2016R1A5A2945889, 2019R1A2B5B02069979, and 2021R1A2C4002158).

## Acknowledgments

The specimens used in this study were provided by the Samsung Medical Center BioBank (20190011 and 20190013).

## Conflict of interest

The authors declare that the research was conducted in the absence of any commercial or financial relationships that could be construed as a potential conflict of interest.

## Publisher’s note

All claims expressed in this article are solely those of the authors and do not necessarily represent those of their affiliated organizations, or those of the publisher, the editors and the reviewers. Any product that may be evaluated in this article, or claim that may be made by its manufacturer, is not guaranteed or endorsed by the publisher.
