# Supplementary material for: Unearthing novel fusions as therapeutic targets in solid tumors using targeted RNA sequencing
Source: Front Oncol. 2022 Aug 10;12:892918. doi: 10.3389/fonc.2022.892918 (PMC9399837; doi:10.3389/fonc.2022.892918)
Supplement: Supplementary file 1 [file DataSheet_1.docx]

Supplementary Material

## Supplementary Figures


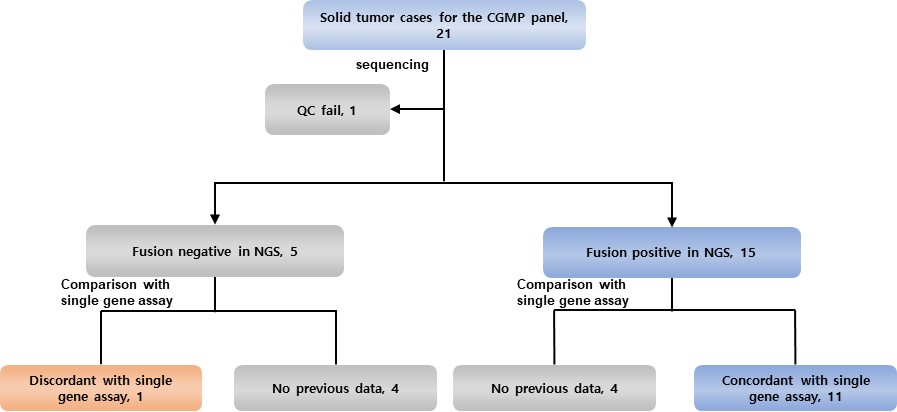


**Supplementary Figure 1.** Overview of NGS panel validation. A total of 21 FFPE samples were sequenced by mixing samples that had already been tested and samples that had not yet been tested. Following sequencing, one sample was lost due to QC failure in the fusion analysis pipeline due to low GSP percentage, but a HEY1-NCOA2 fusion was detected and fusions were found in 15 of the remaining 20 samples. Eleven of the fusion-positive samples were confirmed using traditional assays such as RT-PCR or FISH prior to sequencing, consistent with the sequencing results. One of the five fusion-negative samples was DDIT3-positive by FISH, but was not detected by the panel due to the absence of the DDIT3 gene; the panel was subsequently improved.


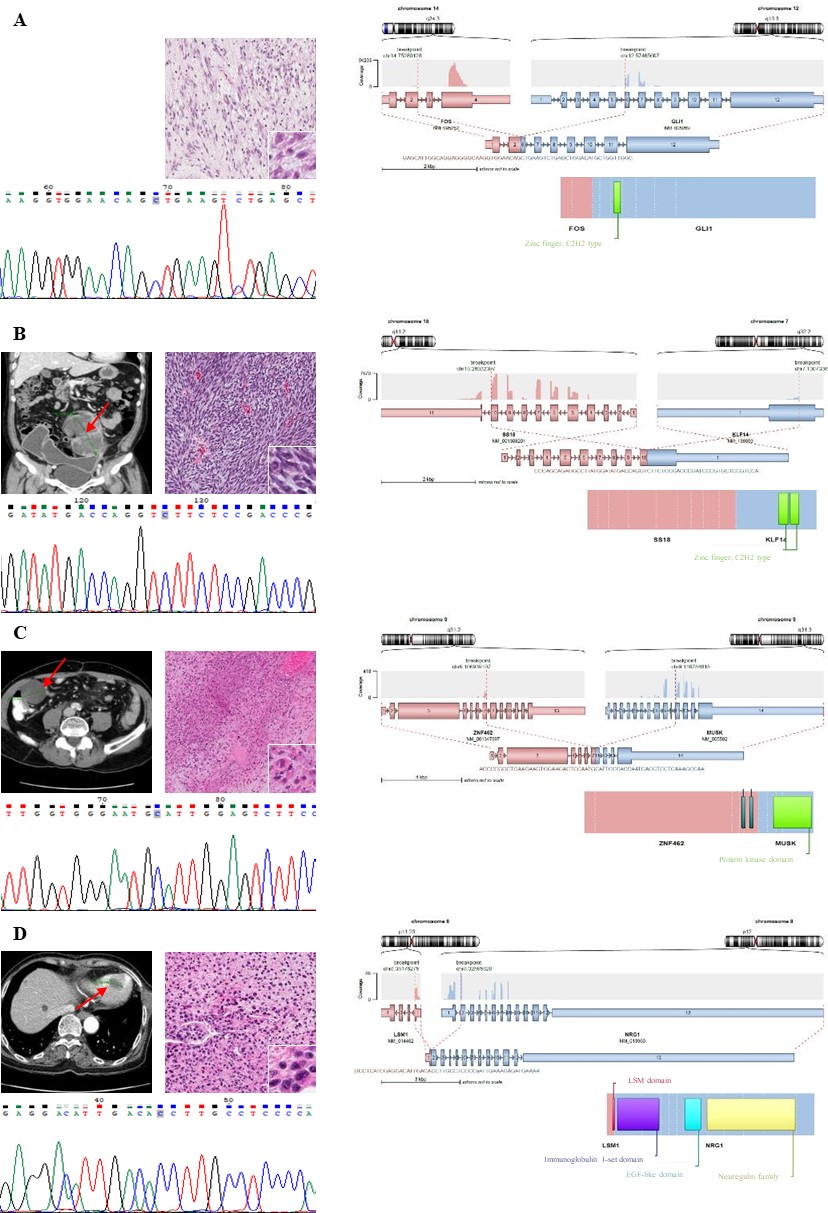


**Supplementary Figure 2**. Other detected novel fusions. (A) H&E staining shows spindle cell proliferation with myxoid stroma. The protein domains of detected FOS-GLI1 fusion shows the Zinc finger domain of GLI1 remained. (B) Computed tomography of abdominopelvic shows a 7.6cm adjacent with left pelvic wall (left) H&E staining shows monotonous spindle cell forming fascicular pattern (right). The protein domains of detected SS18-KLF14 fusion shows the QPGY domain of SS18 remained. (C) CT image of abdomen shows abdominal palpable mass in mesentery of right lower quadrant abdomen (left) H&E staining shows atypically scattered spindle cell in the myxoid background (right). The protein domains of detected ZNF462-MUSK fusion shows the protein kinase domain of MUSK and DNA binding domain of ZNF462 remained. (D) Cardiac sonography displays a mobile mass attached mitral valve leaflet (left) H&E staining shows spindle cells and some scattered pleomorphic cells with focal necrosis (right). The protein domains of detected LSM1-NRG1 fusion. All fusions were confirmed by RT-PCR.


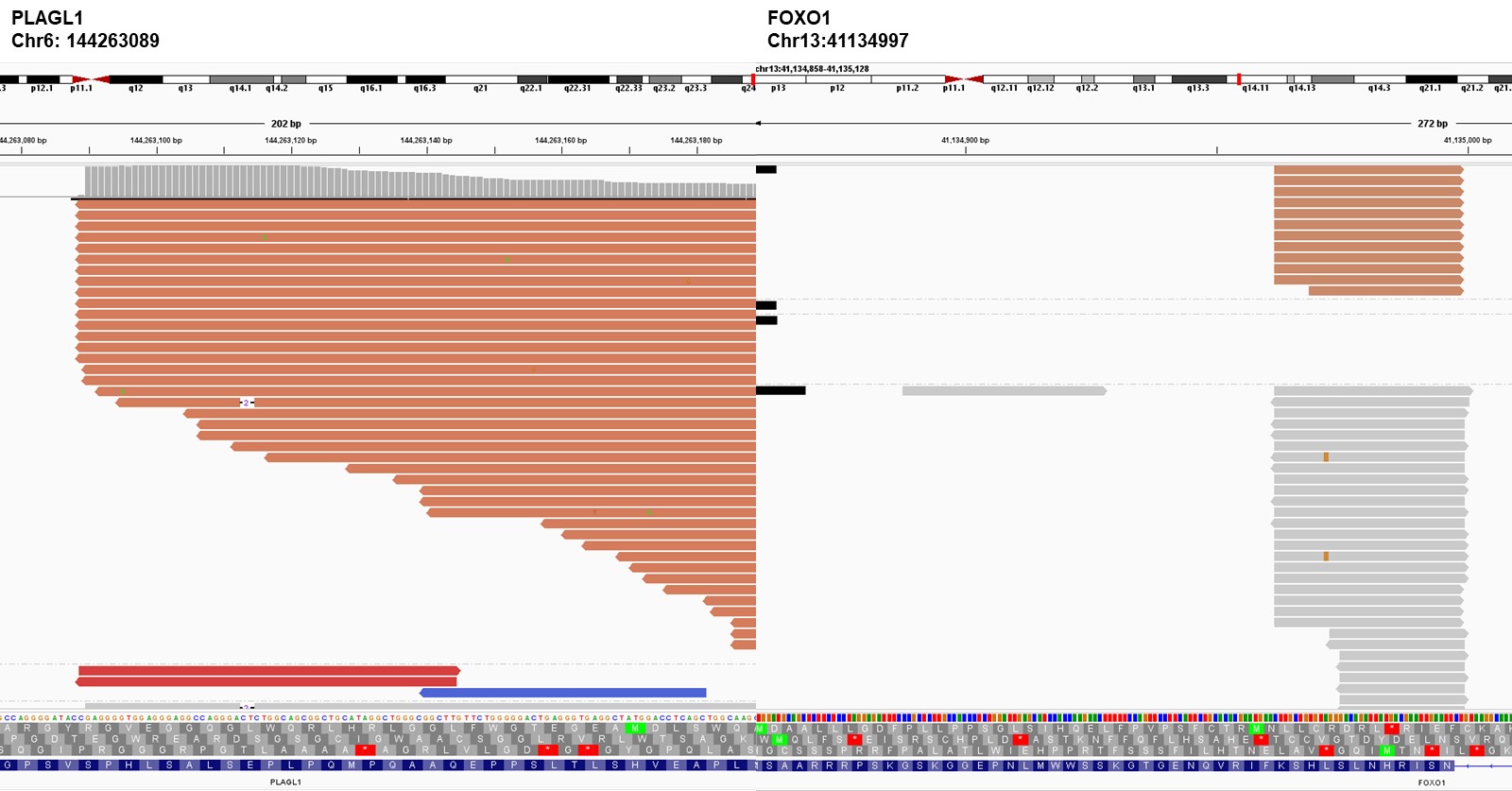

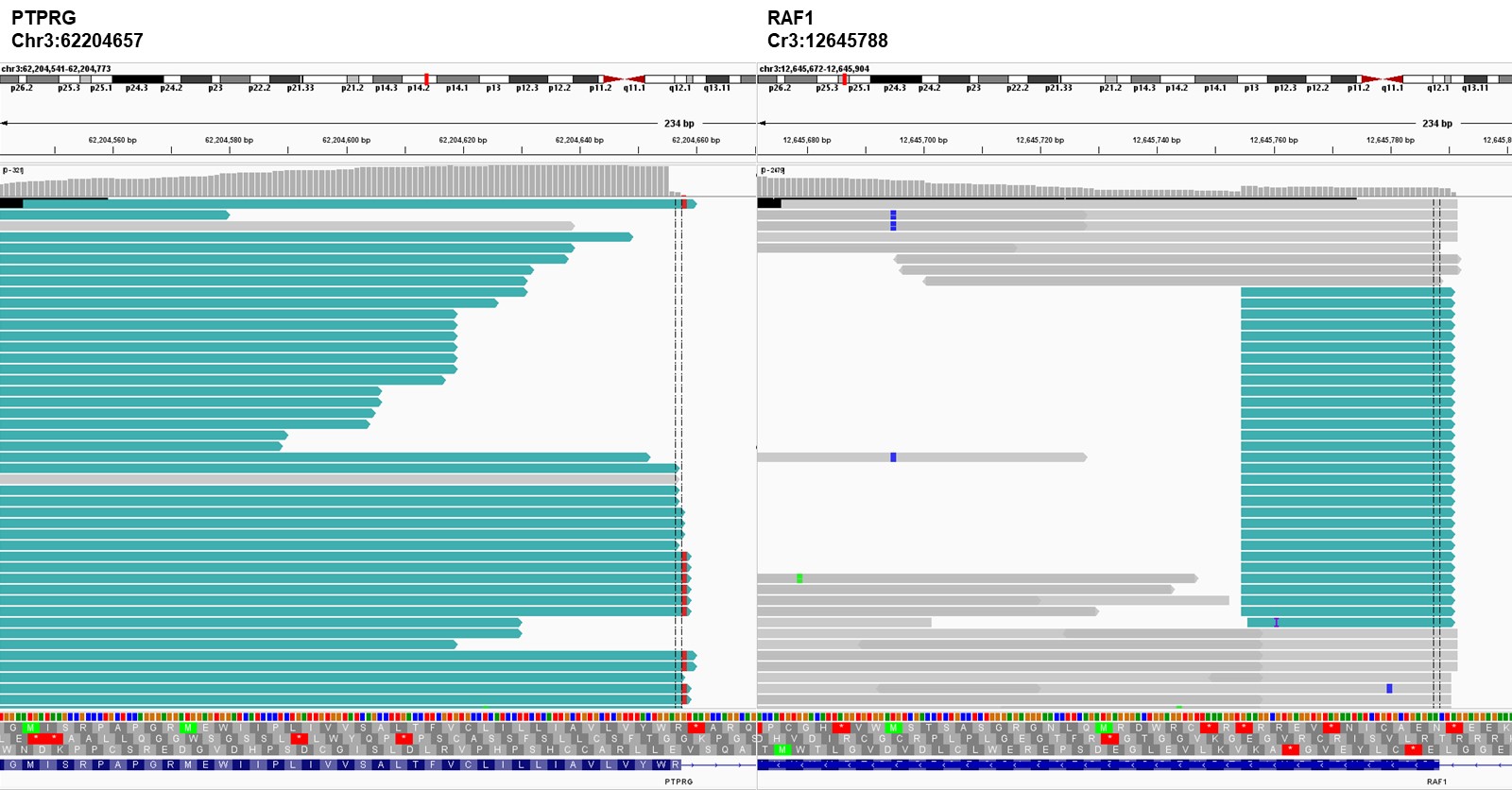

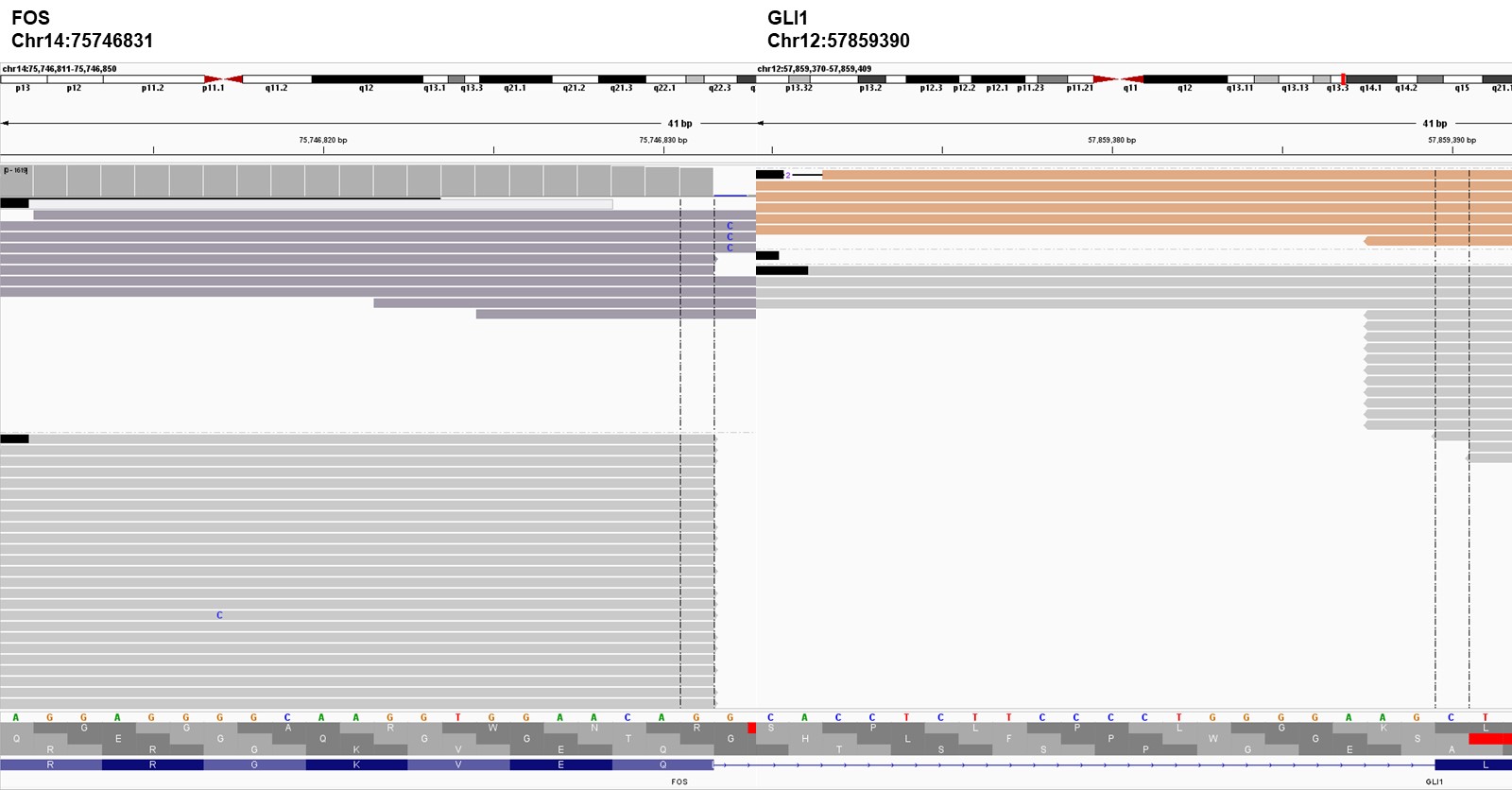

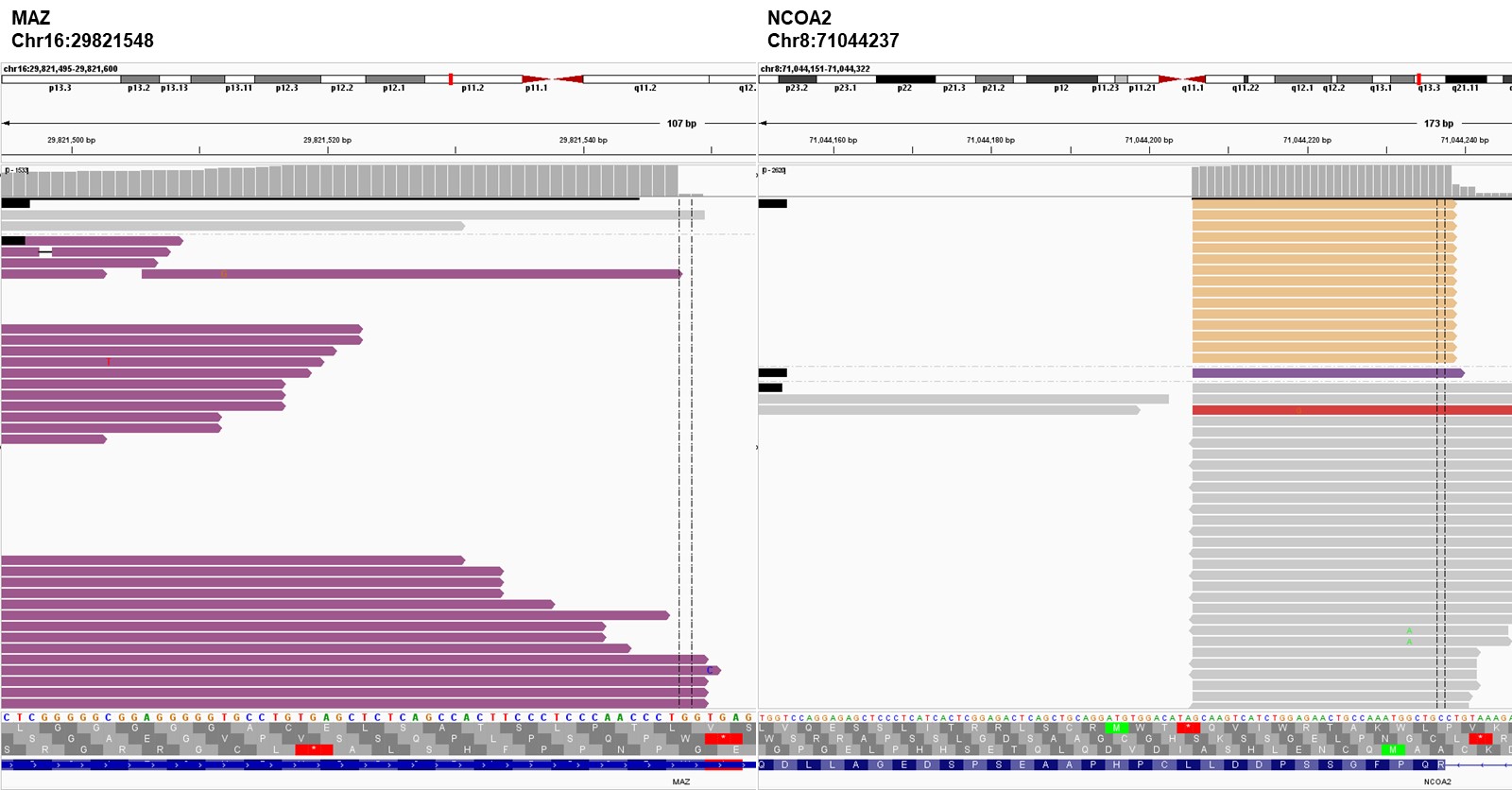


**A**

**B**

**C**

**D**


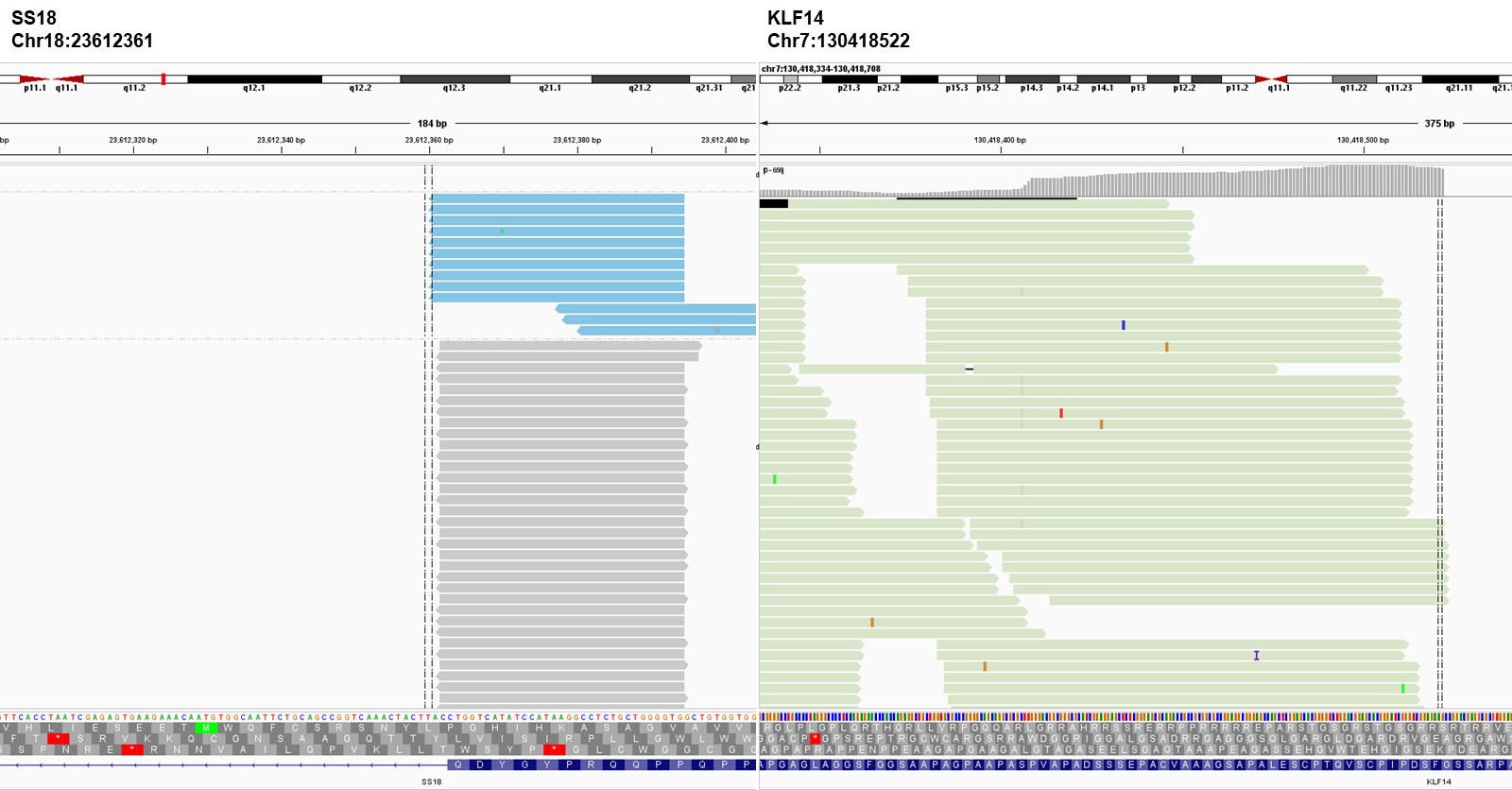

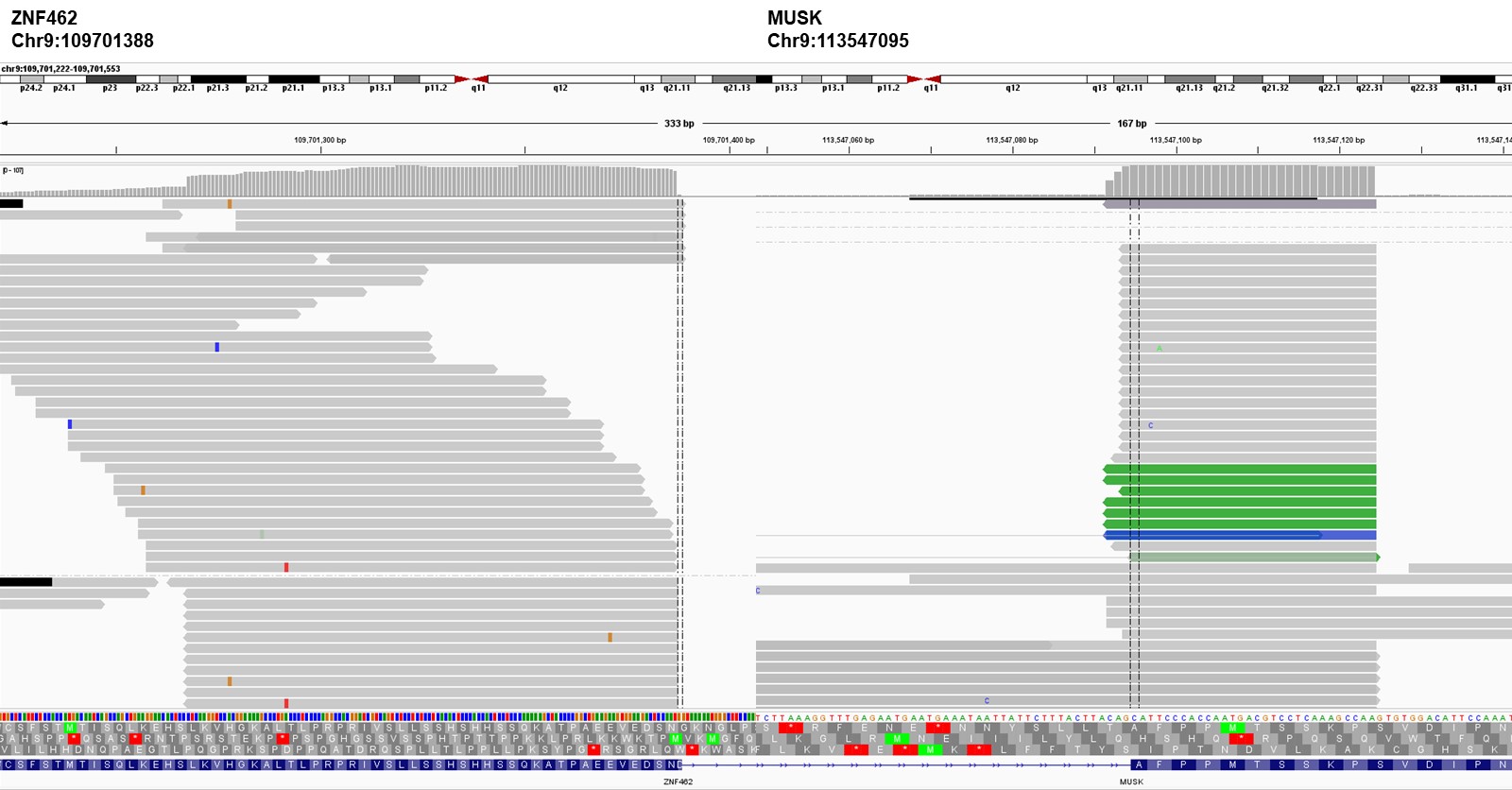

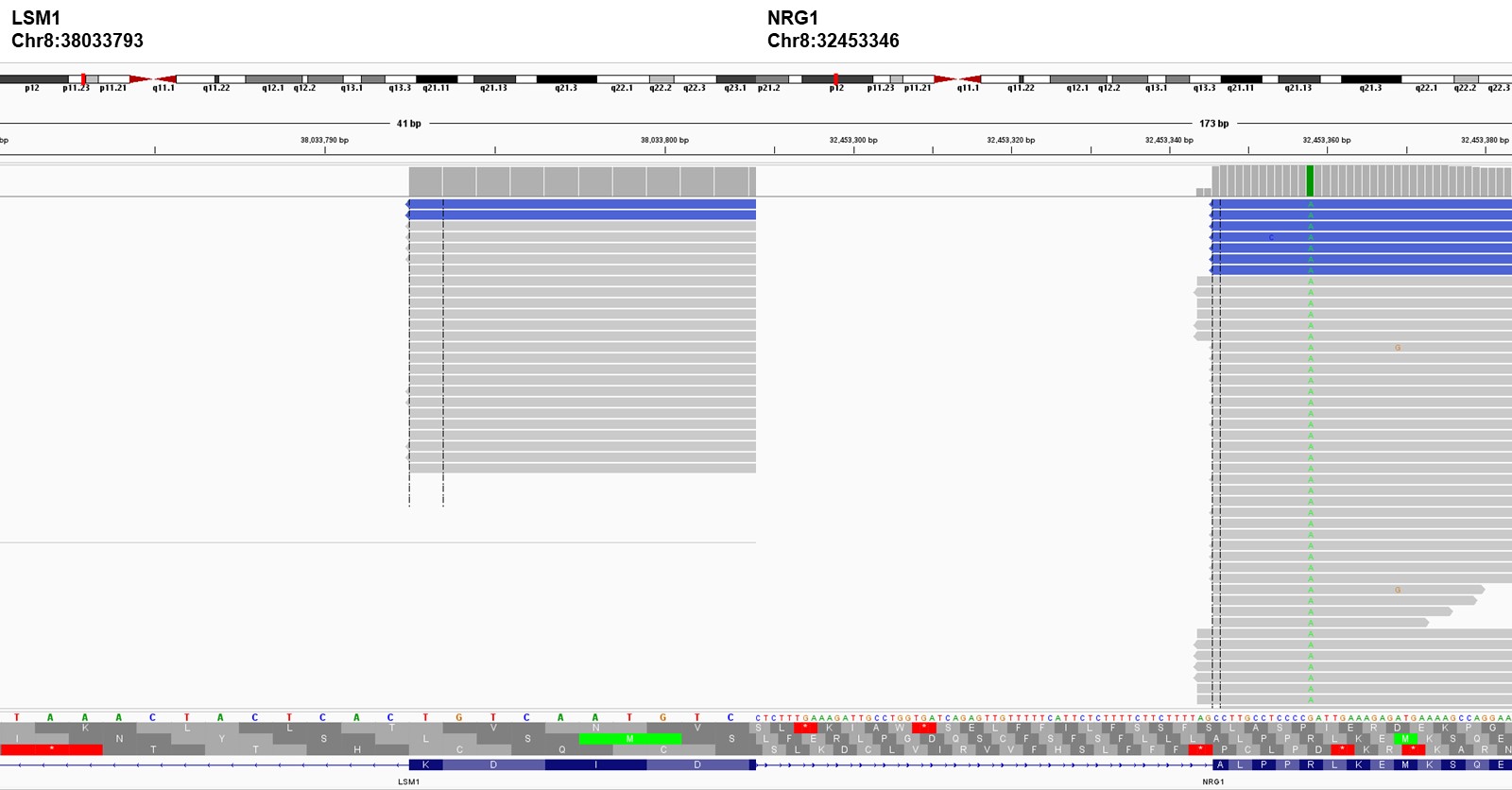

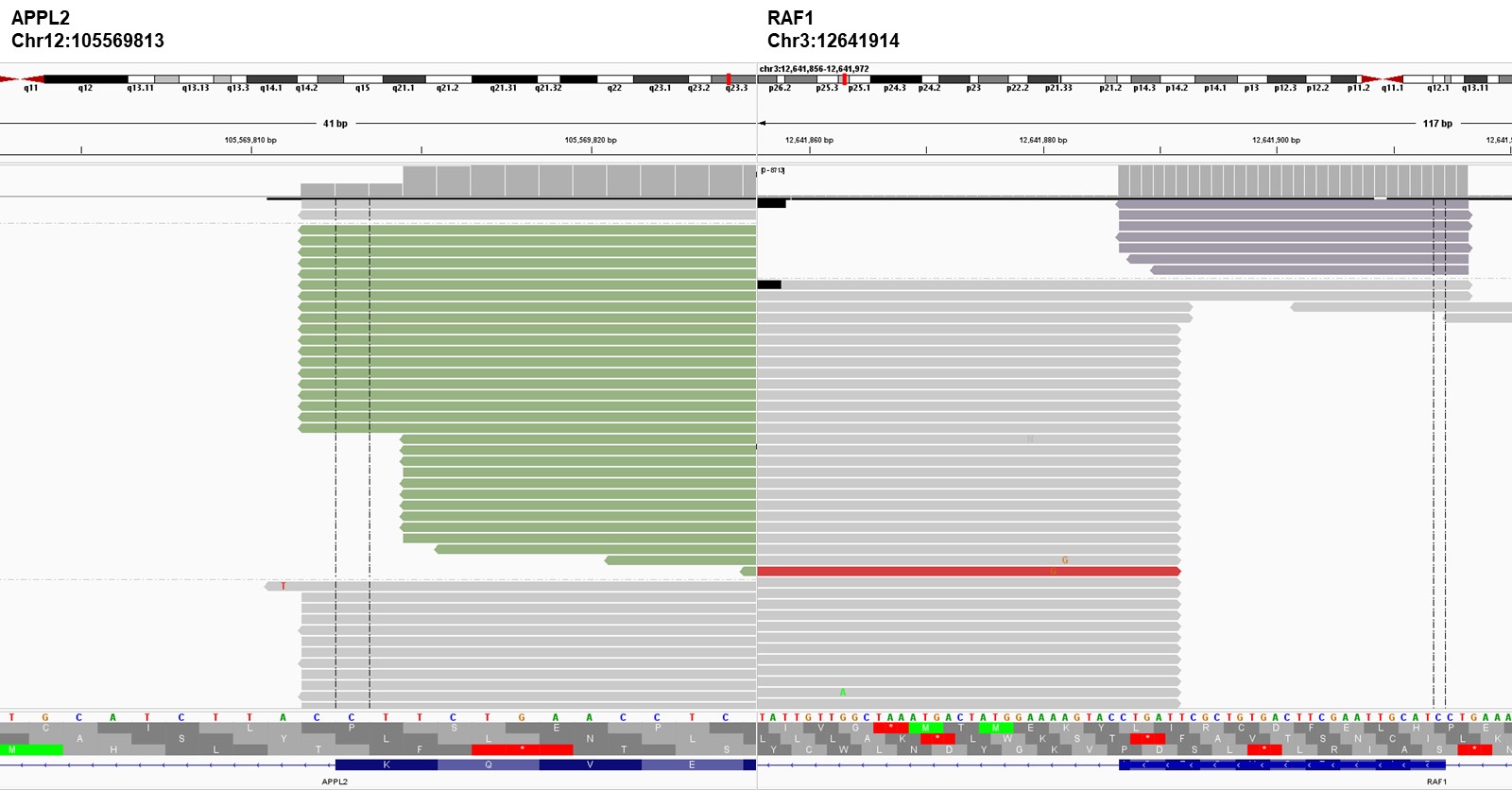
**Supplementary Figure 3**. IGV visualization of fusions. (A) PLAGL1-FOXO1 fusion in AF0033 (B) PTPRG-RAF1 fusion in AF0062 (C) FOS-GLI1 fusion in AF0111 (D) MAZ-NCOA2 fusion in AF0112 (E) SS18-KLF14 fusion in AF140 (F) ZNF462-MUSK fusion in AF171 (G) LSM1-NRG1 fusion in AF197 (H) APPL2-RAF1 fusion in AF215

**E**

**F**

**G**

**H**


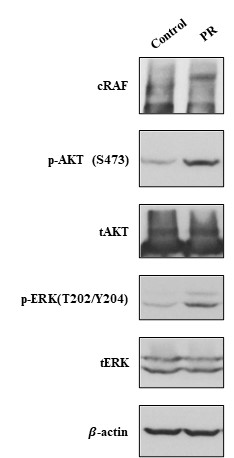


**Supplementary Figure 4.** Western blotting for comparison between NIH3T3 control and PR cells.


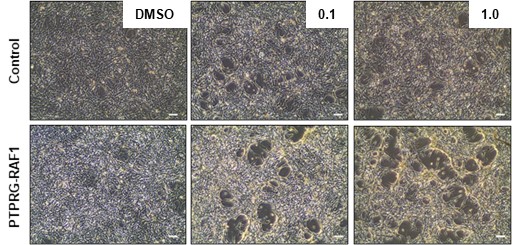


**Supplementary Fig 5.** Proliferation in the presence of 0.1 µM and 1 µM dabrafenib for 48 h.

| **Case number** | **Diagnosis** | **Sequencing** | **Fusions** | **BP** | **FISH** | **IHC** | **RT-PCR** | **Single gene assay** |
| --- | --- | --- | --- | --- | --- | --- | --- | --- |
| AF0001 | metastatic clear cell sarcoma |  | *EWSR1*:CREB1 | chr22:29687604,chr2:208438803 | EWSR + |  |  | Concordant |
| AF0002 | embryonal rhabdomyosarcoma |  |  |  | FOXO - |  |  | No previous report |
| AF0003 | low grade fibromyxoid sarcoma |  | *FUS*:CREB3L2 | chr16:31198133,chr7:137593110 |  |  |  | No previous report |
| AF0004 | Solitary fibrous tumor |  | NAB2:*STAT6* | chr12:57486751,chr12:57501097 |  | STAT6 + |  | Concordant |
| AF0005 | mesenchymal chondrosarcoma | Sample QC X | HEY1:*NCOA2* | chr8:80678886,chr8:71057083 | SS18 - |  |  | No previous report |
| AF0006 | alveolar soft part sarcoma |  | ASPSCR1:*TFE3* | chr17:79954722,chrX:48891766 |  | TFE3 + |  | Concordant |
| AF0007 | desmoplastic small round cell tumor |  | *EWSR1*:WT1 | chr22:29683123,chr11:32414301 | EWSR + |  |  | Concordant |
| AF0008 | alveolar rhabdomyosarcoma |  | PAX3:*FOXO1* | chr2:223084859,chr13:41134997 | FOXO + |  |  | Concordant |
| AF0009 | synovial sarcoma |  | *SS18*:SSX1 | chr18:23612363,chrX:48123217 |  |  | SYT-SSX1 + | Concordant |
| AF0010 | synovial sarcoma |  | *SS18*:SSX1 | chr18:23612363,chrX:48123217 |  |  | SYT-SSX1 + | Concordant |
| AF0011 | chondrosarcoma |  |  |  |  |  |  | No previous report |
| AF0012 | Inflammatory myofibroblastic tumor |  | PPFIBP1:*ALK* | chr12:27809663,chr2:29446394 |  | ALK focal + |  | Concordant |
| AF0013 | Inflammatory myofibroblastic tumor |  | FN1:*ALK* | chr2:216235017,chr2:29448431 | ALK + | ALK + |  | Concordant |
| AF0014 | Ewing sarcoma |  | *EWSR1:ERG* | chr22:29683123,chr21:39764366 |  |  |  | No previous report |
| AF0015 | Ewing sarcoma |  | *EWSR1*:FLI1 | chr22:29683123,chr11:128675261 | EWSR + |  |  | Concordant |
| AF0016 | ALK expressing adenocarcinoma |  | EML4:*ALK* | chr2:42522656,chr2:29446394 |  | ALK + |  | Concordant |
| AF0017 | myxoid liposarcoma |  |  |  | DDIT3 - |  |  | No previous report |
| AF0018 | Endometrial stromal sarcoma |  | *JAZF1*:SUZ12 | chr7:27934839,chr17:30267305 |  |  |  | No previous report |
| AF0019 | Endometrial stromal sarcoma |  |  |  |  |  |  | No previous report |
| AF0020 | myxoid liposarcoma |  |  |  | DDIT3 + |  |  | Discordant |
| AF0021 | Chondrosarcoma |  | COL1A1:*PDGFB* | chr17:48272928,chr22:39631879 |  |  |  | No previous report |

**Supplementary table 1**. The results of primary panel test. Analytic validity of primary panel by comparing to single gene assay results.

| AKT1 | AKT2 | AKT3 | ALK | **AR** | ARHGAP26 | AXL | BCL2 | BCOR | **BCORL1** |
| --- | --- | --- | --- | --- | --- | --- | --- | --- | --- |
| BRAF | BRD3 | BRD4 | CAMTA1 | CCNB3 | CDK2 | CIC | CNOT2 | **COL6A3** | CSF1 |
| **DDIT3** | DNAJB1 | EGFR | EPC1 | ERBB2 | ERBB4 | ERG | ESR1 | ESRRA | ETV1 |
| ETV4 | ETV5 | ETV6 | EWSR1 | FGFR1 | FGFR2 | FGFR3 | FGR | FOS | FOSB |
| FOXO1 | FUS | GLI1 | HMGA2 | INSR | **JAK3** | JAZF1 | **KIT** | MAML2 | MAST1 |
| MEAF2 | MEAF6 | MET | **MGEA5** | MKL2 | **MN1** | MSMB | MUSK | MYB | MYBL1 |
| **NCOA**1 | NCOA2 | NOTCH1 | NOTCH2 | **NR4A3** | NRG1 | NTRK1 | NTRK2 | NTRK3 | NUAK1 |
| NUMBL | NUTM1 | **NUTM2A** | **NUTM2B** | PDGFB | PDGFRA | PDGFRB | **PHF1** | PIK3CA | PKN1 |
| PLAG1 | PPARG | PRKACA | PRKCA | PRKCB | **PTCH1** | **PTPN1** | RAF1 | RELA | RET |
| ROS1 | RSPO2 | RSPO3 | SS18 | STAT6 | TAF15 | TCF12 | TERT | TFE3 | TFEB |
| TFG | THADA | TMPRSS2 | USP6 | **VGLL3** | **YAP1** | YWHAE |  |  |  |

**Supplementary Table 2**. Genes included in the Cancer Gene-fusion by Multiplex PCR (CGMP), panel. The 107 genes are present in fusion databases.

*Genes marked with bold are added after validation of primary panel.

**Supplementary Table 3.** Sample information. The group was classified into 14 groups. 1. Adenocarcinoma 2. Carcinoma, a known lineage 3. Carcinoma, a known fusion 4. Carcinoma, unknown lineage; undifferentiated carcinoma 5. Uterine malignancy 6. Sarcoma, unknown lineage; undifferentiated sarcoma 7. Sarcoma, known fusion 8. Sarcoma, myogenic lineage without known fusion 9. Sarcoma, fibroblastic lineage without known fusion 10. Sarcoma, neurogenic lineage without known fusion 11. Sarcoma, chondroid or bone lineage without known fusion 12. Sarcoma, lipogenic lineage without known fusion 13. Sarcoma, vascular or perivascular lineage without known fusion 14. Other malignancy. (Separated file)

| **Group No.** | **Group** | **Diagnosis** |
| --- | --- | --- |
| 1 | Adenocarcinoma |  |
| 2 | Carcinoma, known lineage |  |
| 3 | Carcinoma, known fusion | NUT carcinoma |
| 4 | Carcinoma, unknown lineage; undifferentiated carcinoma |  |
| 5 | Uterine malignancy |  |
| 6 | Sarcoma, unknown lineage; undifferentiated sarcoma | Epithelioid sarcoma |
|  |  | Intimal sarcoma |
|  |  | PEComa |
|  |  | Epithelioid angiomyolipoma |
|  |  | Myoepithelioma |
| 7 | Sarcoma, known fusion | Synovial sarcoma |
|  |  | Sclerosing epithelioid fibrosarcoma |
|  |  | Clear cell tumor |
|  |  | Ewing sarcoma |
|  |  | Solitary fibrous tumor |
|  |  | Embryonal rhabdomyosarcoma |
|  |  | Dermatofibrosarcoma protuberans |
|  |  | Desmoplastic small round cell tumor |
|  |  | Extraskeletal myxoid chondrosarcoma |
|  |  | Mesenchymal chondrosarcoma |
|  |  | Alveolar soft part sarcoma |
|  |  | Alveolar rhabdomyosarcoma |
|  |  | Low grade fibromyxoid sarcoma |
| 8 | Sarcoma, myogenic lineage without known fusion |  |
| 9 | Sarcoma, fibroblastic lineage without known fusion |  |
| 10 | Sarcoma, neurogenic lineage without known fusion |  |
| 11 | Sarcoma, chondroid or bone lineage without known fusion |  |
| 12 | Sarcoma, lipogenic lineage without known fusion |  |
| 13 | Sarcoma, vascular or perivascular lineage without known fusion |  |
| 14 | Other malignancy | Wilms tumor |
|  |  | Mesothelioma |
|  |  | Myeloid sarcoma |
|  |  | Embryonal tumor |

**Supplementary Table 4.** Diagnostic names by type of cancer groups.

| **Gene** | **Transcript ID** | **Primer sequence** |
| --- | --- | --- |
| PLAGL1 F | NM_001080951.3 | 5'-GGTCCATAGCCTCACCCTCA-3' |
| FOXO1 R | NM_002015.4 | 5'-TGCACACGAATGAACTTGCT-3' |
| PTPRG F | NM_002841.4 | 5'-TGGTATCAGCCTTGACCTTCG-3' |
| RAF1 R | NM_001354689.3 | 5'-GATGAGGGACTGGAGGTGTT-3' |
| FOS F | NM_005252.4 | 5'-CGTTGTGAAGACCATGACAGGA-3' |
| GLI1 R | NM_005269.3 | 5'-TTGGGGCTGGACATATCACCTT-3' |
| MAZ F | NM_001042539.3 | 5'-AGCTCTCAGCCACTTCCCTC-3' |
| NCOA2 R | NM_001321703.2 | 5'-AATTCCGCAAGGCCAGATACAG -3' |
| APPL2 F | NM_001251905.2 | 5’-GAAAGCAACTCAGAAGGCGA-3’ |
| RAF1 R | NM_001354689.3 | 5’-TACTGGACAGGGCTGAAGGT-3’ |
| SS18 | NM_001308201.2 | 5’- CTACGGTCCTTCACAGGGTG -3’ |
| KLF14 | NM_138693.4 | 5’- GACTTGAGGTGCGACGACTT -3’ |
| ZNF462 | NM_001347997.2 | 5’-GATCGTCAGTCTCCTCTCCTC-3’ |
| MUSK | NM_005592.5 | 5’-GTCATGGAGTATGTAGGTGAGACA-3’ |
| LSM1 | NM_014462.3 | 5’-GCATTGCAGCATTATTTCAGTTCA-3’ |
| NRG1 | NM_013960.5 | 5’-TGGTTTCACACCGAAGGACT-3’ |

**Supplementary Table 5**. Novel fusion PCR primers.
